# Dopaminergic treatment strategies for people with Parkinson’s disease in Europe: a retrospective analysis of PRISM trial data

**DOI:** 10.1007/s10072-023-06888-5

**Published:** 2023-06-13

**Authors:** Lara Hansen, Victoria Witzig, Jörg B. Schulz, Florian Holtbernd

**Affiliations:** 1 Department of Neurology, RWTH Aachen University, Pauwelsstr. 30, 52074 Aachen, Germany; 2 JARA-BRAIN Institute Molecular Neuroscience and Neuroimaging, Juelich Research Center GmbH and RWTH Aachen University, Aachen, Germany; 3 Juelich Research Center, Institutes of Neuroscience and Medicine (INM-4, INM-11), Juelich, Germany

**Keywords:** Parkinson’s disease, Levodopa, Prescription practice, Prescription pattern

## Abstract

**Background:**

Levodopa (LD) is the most effective drug to treat Parkinson’s disease (PD). The recently concluded multinational Parkinson’s Real-World Impact Assessment (PRISM) trial revealed highly variable prescription patterns of LD monotherapy across six European countries. The reasons remain unclear.

**Methods:**

In this post hoc analysis of PRISM trial data, we used multivariate logistic regression analysis to identify socio-economic factors affecting prescription practice. We applied receiver-operated characteristics and split sample validation to test model accuracy to predict treatment class (LD monotherapy vs. all other treatments).

**Results:**

Subject age, disease duration, and country of residence were significant predictors of treatment class. The chance of receiving LD monotherapy increased by 6.9% per year of age. In contrast, longer disease duration reduced the likelihood of receiving LD monotherapy by 9.7% per year. Compared to the other countries, PD patients in Germany were 67.1% less likely and their counterparts in the UK 86.8% more likely to receive an LD monotherapy. The model classification accuracy of treatment class assignment was 80.1%. The area under the curve to predict treatment condition was 0.758 (95% CI [0.715, 0.802]). Split sample validation revealed poor sensitivity (36.6%), but excellent specificity (92.7%) to predict treatment class.

**Conclusion:**

The relative lack of socio-economic variables affecting prescription practice in the study sample and limited model accuracy to predict treatment class suggest the presence of additional, country-specific factors affecting prescription patterns that were not assessed in the PRISM trial. Our findings indicate that physicians still avoid prescribing LD monotherapy to younger PD patients.

**Supplementary Information:**

The online version contains supplementary material available at 10.1007/s10072-023-06888-5.

## Introduction

Parkinson’s disease (PD) is a progressive neurodegenerative disease with an estimated prevalence of 1% in people above 60 years, and 3% in people older than 80 years [1]. The defining motor symptoms are brady-/hypokinesia, rigidity, tremor, and postural instability [2]. The most potent drug available to treat the motor symptoms of PD is levodopa (LD). LD improves activities of daily living, is cost-efficient, and has fewer side effects compared with dopamine agonists (DA) or monoamine oxidase-B inhibitors (MAOB-I) [3–5]. However, compared with DA and MAOB-I, LD treatment bears a higher risk for the early development of dyskinesias [6]. This is especially true for younger patients [6]. Thus, it has been a long-standing dogma that LD should be avoided in the intial treatment of PD patients < 60 years of age [7]. Even though the majority of currently available treatment guidelines recommend DA, MAOB-I, and LD as equally suitable treatment options in early PD, no matter of subject age [8, 9], epidemiological studies show substantial differences in international prescription practices [10]. Along these lines, a recently published multinational observational study involving more than 800 PD patients from six European countries, the Parkinson’s Real-World Impact Assessment (PRISM) trial, confirmed a highly variable usage of dopaminergic drugs to treat PD [11]. In particular, there was a wide range of the prevalence of LD monotherapy, ranging from 8.3% in Germany to 38.3% in the UK [11]. The reasons for these substantial differences remain elusive. The aim of this retrospective analysis of PRISM trial data was to assess the impact of demographic, economic, and medical factors on drug prescription practice for people with PD in Europe.

## Materials and methods

### The PRISM study

The PRISM study was a multinational, observational, cross-sectional survey designed by an international scientific committee in collaboration with the Cure Parkinson’s Trust (a UK-based research-driven charity) [11]. Patients were recruited from six different European countries: France, Germany, Italy, Portugal, Spain, and the UK. In total, 861 PD patients and their carers were recruited (63 (7.3%) patients from France, 92 (10.7%) from Germany, 264 (30.7%) from Italy, 80 (9.3%) from Portugal, 149 (17.3%) from Spain, and 213 (24.7%) from the UK). Socio-economic and demographic data were assessed using structured online questionnaires. The Parkinson’s Disease Questionnaire (PDQ-39) [12] and Parkinson’s Disease non-motor symptoms questionnaire (PD-NMS) [13] were used to assess quality of life (QoL) and non-motor symptoms. All participants were categorized according to the current PD treatment, including LD monotherapy and several drug combinations (Table 1). All data of the PRISM trial have been published descriptively, and no statistical analyses were performed. Details of the study design are stated in the original publication [11]. The PRISM trail was conducted according to ethical standards as mandated by the local authorities (NHS England Health Research Authority) [11].

**Table 1:** Treatment condition of study participants. *LD*, levodopa; *DA*, dopamine agonist; *MAOB-I*, monoamine oxidase-B inhibitor; *COMT-I*, catechol-O-methyltransferase inhibitor

Drug combinations	*n*	%
LD	172	20.0
DA	32	3.7
LD + DA	108	12.5
LD + MAOB-I	72	8.4
DA + MAOB-I	28	3.3
LD + DA + MAOB-I	113	13.1
LD + DA + MAOB-I + COMT-I	32	3.7
LD + DA + COMT-I	26	3.0
LD + COMT-I	21	2.4
MAOB-I	14	1.6
Other treatment	133	15.4
No treatment	39	4.5
Missing data	71	8.2

### Data assessment and statistical analyses

Information about the current treatment modality was missing in 71/861 patients. Thus, 790 PD patients (59 (7.5%) patients from France, 84 (10.6%) from Germany, 235 (29.7%) from Italy, 75 (9.5%) from Portugal, 141 (17.8%) from Spain, and 196 (24.8%) from the UK) were included in the analysis. We screened the PRISM database for demographic, economic, and medical factors that potentially may impact prescription practice of PD medication. The following variables were selected: current age, gender, age at diagnosis, disease duration, country of residence, PDQ-39 total score, NMS total score, presence of comorbidities (yes vs. no), number of comorbidities, presence of dementia (yes vs. no), specialist appointment in the last 12 months (yes vs. no), one-way travel distance between home and specialist (miles or km), out-of-pocket costs for prescription drugs (yes vs. no), and emergency room or hospital visit in the last 12 months (yes vs. no). Missing data for all variables are summarized in Table S1. First, we used ANOVA or Chi-square tests where appropriate, to explore group differences across the six European countries for each variable. For ANOVA, we used post hoc Bonferroni tests for pairwise comparisons. A *p*-value of < 0.05 was deemed significant. For the Chi-square test, we computed adjusted standardized residuals for post hoc testing. Adjusted residuals were converted into Bonferroni corrected *p*-values to account for multiple testing [15].

We next designed a univariate binary logistic regression model using “LD monotherapy” as dependent and each of the abovementioned variables as independent factor. To determine the effect of the factor “country” on the prevalence of LD monotherapy, all patients were classified according to their country of residence and were compared to all other patients from the respective other 5 countries, resulting in six dummy variables [16]. Only variables achieving a threshold of *p* < 0.25 in the univariate analyses were used in the final multivariate binary logistic regression to avoid over- or underestimation of true effects [17]. To assess the predictive accuracy of the model, we employed receiver operating characteristics (ROC). In addition to ROC analyses, we applied a split sample validation to test the predictive accuracy of the model by randomly dividing the entire dataset into a training sample (75% of study subjects) and test sample (25% of study subjects) [18]. Statistical analyses were performed using SPSS 27 (IBM Corp., Armonk, NY, USA).

### Data availability

Data used in the preparation of this article were obtained from the Parkinson’s Real World Impact assesSMent (PRISM) database (http://www.prism.bial.com).

## Results

### Comparison of demographic, economic, and medical factors across countries

**Fig. 1:**
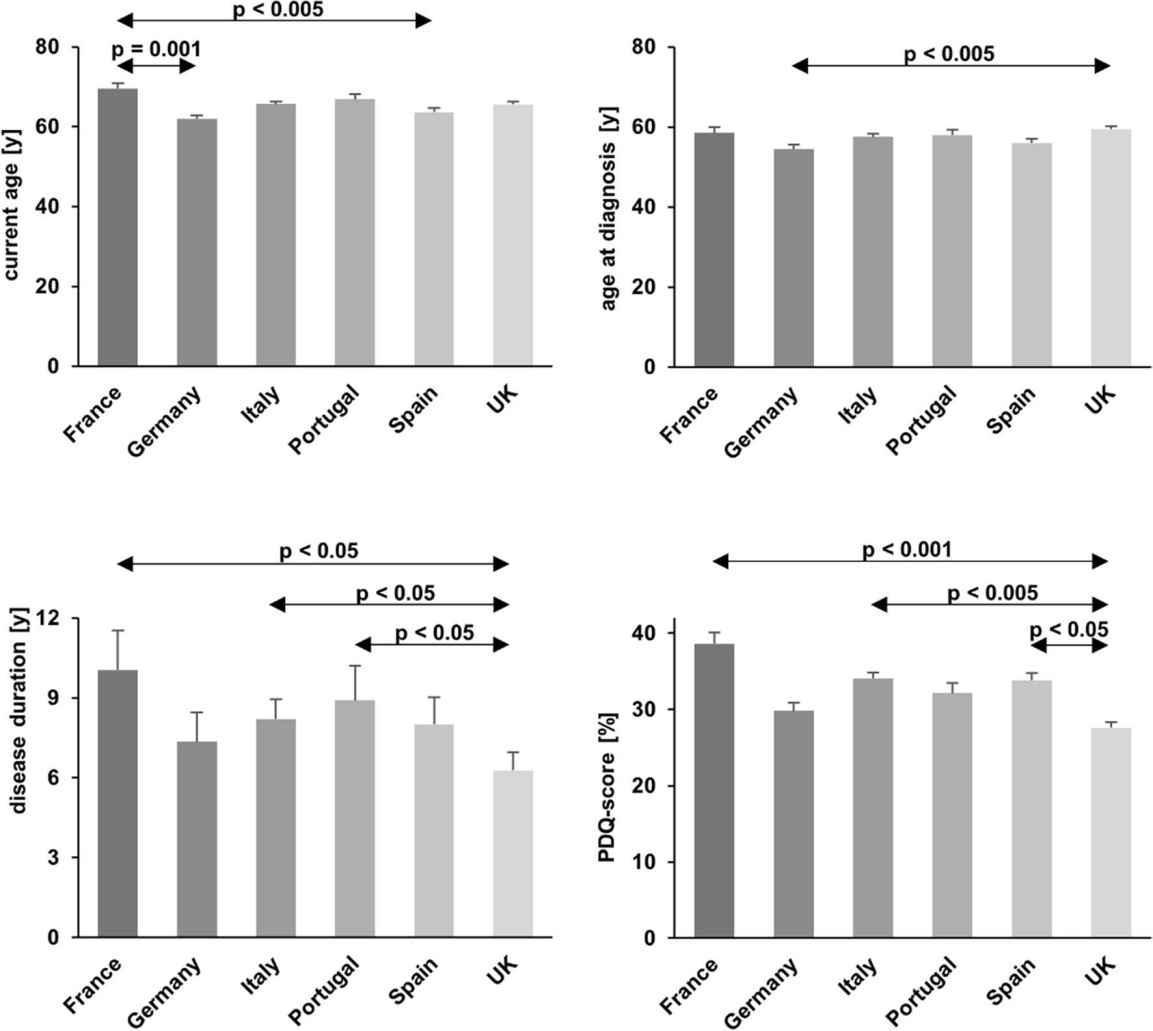
Results of significant ANOVA of demographic and behavioral data across countries. PDQ-39, Parkinson’s disease questionnaire total score; PD-NMS, Parkinson’s disease non-motor symptom score. Bars represent group means, error bars the SEM

**Fig. 2:**
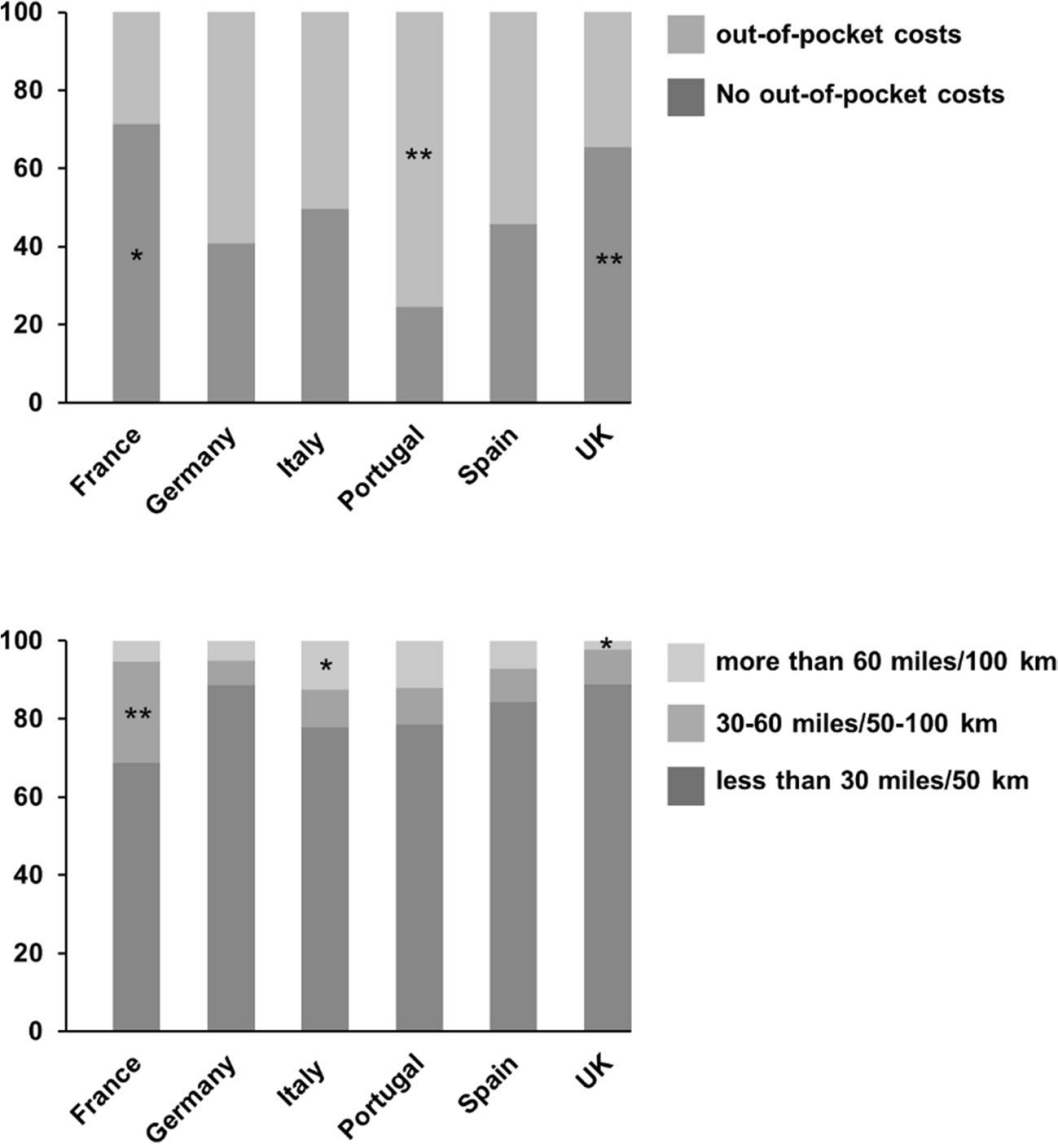
Results of significant Chi-square tests of demographic and behavioral data across countries. Bars represent group means, error bars the SEM; * *p* < 0.05, ** *p* < 0.001, post hoc Bonferroni corrected *p*-values (see text for details)

We found significant differences of age (*F(5,778)* = *4.763, p* < *0.001*), age at diagnosis (*F(5,750)* = *3.349, p* = *0.005*), disease duration (*F(5,748)* = *4.224, p* < *0.001*), total PDQ-39 score (*F(5,783)* = *4.847, p* < *0.001*), PD-NMS-score (*F(5,579)* = *2,917, p* = *0.013)*, one-way travel distance between home and specialist (*χ*^*2*^*(10)* = *37.787, p* < *0.001*), and out-of-pocket costs (*χ*^*2*^*(5)* = *48.231, p* < *0.001*) among the six countries (Fig. 1 and Fig. 2). The number of patients below the age of 60 was significantly higher in Germany and Spain, and lower in France compared to the respective other countries (*χ*^*2*^*(5)* = *29.763, p* < *0.001*). There were no differences of the presence or absence of comorbidities (*p* = 0.069), number of comorbidities (*p* = 0.325), presence or absence of dementia (*p* = 0.183), specialist appointment in the last 12 months (*p* = 0.320), or emergency or hospital visits in the last 12 months (*p* = 0.775) across countries. Results of post hoc tests are summarized in Tables 2 and 3, respectively.

**Table 2:** Post hoc group-wise comparisons of ANOVAs of demographical and behavioral data among all countries. Significant findings (*p* < 0.05) are printed in italics. The mean differences were calculated based on the country listed first

		*p*-value	Mean difference
Age	*France vs. Germany* France vs. Italy France vs. Portugal *France vs. Spain* France vs. UK Germany vs. Italy Germany vs. Portugal Germany vs. Spain Germany vs. UK Italy vs. Portugal Italy vs. Spain Italy vs. UK Portugal vs. Spain Portugal vs. UK Spain vs. UK	< *0.001* 0.184 1.000 < *0.005* 0.188 0.081 0.055 1.000 0.116 1.000 0.927 1.000 0.478 1.000 1.000	*7.5 y* 3.8 y 2.7 y *6.0 y* 3.9 y − 3.7 y − 4.8 y − 1.6 y − 3.6 y − 1.1 y 2.1 y 0.1 y 3.2 y 1.2 y − 2.0 y
Age at diagnosis	France vs. Germany France vs. Italy France vs. Portugal France vs. Spain France vs. UK Germany vs. Italy Germany vs. Portugal Germany vs. Spain *Germany vs. UK* Italy vs. Portugal Italy vs. Spain Italy vs. UK Portugal vs. Spain Portugal vs. UK Spain vs. UK	0.532 1.000 1.000 1.000 1.000 0.314 0.540 1.000 < *0.006* 1.000 1.000 1.000 1.000 1.000 0.058	4.1 y 0.9 y 0.5 y 2.6 y − 1.0 y − 3.2 y − 3.6 y − 1.6 y * − 5.1 y* − 0.4 y 1.6 y − 1.9 y 2.0 y − 1.5 y − 3.5 y
Disease duration	France vs. Germany France vs. Italy France vs. Portugal France vs. Spain *France vs. UK* Germany vs. Italy Germany vs. Portugal Germany vs. Spain Germany vs. UK Italy vs. Portugal Italy vs. Spain *Italy vs. UK* Portugal vs. Spain *Portugal vs. UK* Spain vs. UK	0.290 1.000 1.000 0.857 < *0.004* 1.000 1.000 1.000 1.000 1.000 1.000 < *0.03* 1.000 < *0.04* 0.232	2.7 y 1.9 y 1.1 y 2.0 y *3.8 y* − 0.8 y − 1.6 y − 0.7 y 1.1 y − 0.7 y 0.2 y *1.9 y* 0.9 y *2.6 y* 1.7 y
Total PDQ-39 score	France vs. Germany France vs. Italy France vs. Portugal France vs. Spain *France vs. UK* Germany vs. Italy Germany vs. Portugal Germany vs. Spain Germany vs. UK Italy vs. Portugal Italy vs. Spain *Italy vs. UK* Portugal vs. Spain Portugal vs. UK *Spain vs. UK*	0.075 1.000 0.664 1.000 < *0.001* 1.000 1.000 1.000 1.000 1.000 1.000 < *0.005* 1.000 1.000 < *0.04*	8.8% 4.5% 6.5% 4.8% *11.0%* − 4.2% − 2.3% − 4.0% 2.2% 1.9% 0.3% *6.5%* − 1.6% 4.5% *6.2%*
Total NMS score	France vs. Germany France vs. Italy France vs. Portugal France vs. Spain France vs. UK Germany vs. Italy Germany vs. Portugal Germany vs. Spain Germany vs. UK Italy vs. Portugal Italy vs. Spain Italy vs. UK Portugal vs. Spain Portugal vs. UK Spain vs. UK	0.559 1.000 1.000 1.000 0.194 1.000 1.000 0.458 1.000 1.000 1.000 0.262 1.000 1.000 0.055	2.6 1.2 2.0 0.5 2.7 − 1.4 − 0.6 − 2.1 0.1 0.9 − 0.7 1.6 − 1.5 0.7 2.2

**Table 3:** Post hoc Bonferroni corrected *p*-values of adjusted standardized residuals of Chi-square tests of demographical data among countries. Significant findings (*p* < 0.05) are printed in italics. Only one category is displayed for dichotomous variables

	Country	Category	Adjusted residuals	Bonferroni corrected *p*-values
One-way travel distance home to specialist	France Germany Italy Portugal Spain UK	< *30miles/50 km* *30–60miles/50–100 km* > 60miles/100 km < 30miles/50 km 30–60miles/50–100 km > 60miles/100 km < *30miles/50 km* 30–60miles/50–100 km > *60miles/100 km* < 30miles/50 km 30–60miles/50–100 km > 60miles/100 km < 30miles/50 km 30–60miles/50–100 km > 60miles/100 km < *30miles/50 km* 30–60miles/50–100 km > *60miles/100 km*	* − 2.79* *4.17* − 0.72 1.58 − 1.19 − 0.92 * − 2.08* − 0.34 *3.37* − 0.88 − 0.21 1.52 0.70 − 0.67 − 0.25 *2.77* − 0.60 * − 3.30*	0.084 < *0.001* 1.000 1.000 1.000 1.000 0.600 1.000 < *0.02* 1.000 1.000 1.000 1.000 1.000 1.000 0.090 1.000 < *0.02*
Out of pocket costs for prescription drugs	*France* Germany Italy *Portugal* Spain *UK*	*Yes* Yes Yes *Yes* Yes *Yes*	* − 2.9* 1.9 0.5 *4.7* 1.4 * − 4.6*	< *0.05* 0.689 1.000 < *0.001* 1.000 < *0.001*
Age categorized in over and under 60 years	France Germany Italy Portugal Spain UK	< 60 years < 60 years < 60 years < 60 years < 60 years < 60 years	* − 3.4* *3.2* − 0.6 − 1.8 *2.8* − 0.8	< *0.008* < *0.02* 1.000 0.862 0.061 1.000

### Effect of socio-economic variables on prescription patterns

In the univariate logistic model, age, age at onset, disease duration, presence of comorbidities, and country achieved the cut-off of *p* < 0.25. Because of a strong correlation of current age and age at onset (*r* = 0.822, *p* < 0.001, Pearson correlation coefficient), we excluded the latter from the final model. The prevalence of LD monotherapy in the overall study population was 22% (172 of 790 individuals). The accuracy of classification (LD monotherapy vs. all other treatments) was 80.1%, with a sensitivity of 20.4% and a specificity of 97.1%. The positive and negative predictive values were 0.665 and 0.812, respectively.

We observed a significant effect of subject age (OR 1.069 (95% CI [1.048, 1.092]); i.e., the chance of receiving LD monotherapy increased by 6.9% per year of age. The likelihood of receiving an LD monotherapy decreased by 9.7% per year after disease onset (OR = 0.903 (95% CI [0.868, 0.939]). Finally, we observed a significant effect of country. Compared with the respective other five countries, patients living in the UK were 86.3% more likely to receive an LD monotherapy (OR = 1.863 (95% CI [1.083, 3.206]). In contrast, patients with PD living in Germany were 67.1% (OR = 0.329 (95% CI [0.132, 0.819]) less likely to receive this therapy. We did not observe significant effects for the remaining countries (Italy, Spain, Portugal, France; *p* > 0.05). There was no effect of the presence of comorbidities (*p* = 0.923) on the likelihood of receiving an LD monotherapy.

### Model accuracy to predict treatment condition

**Fig. 3:**
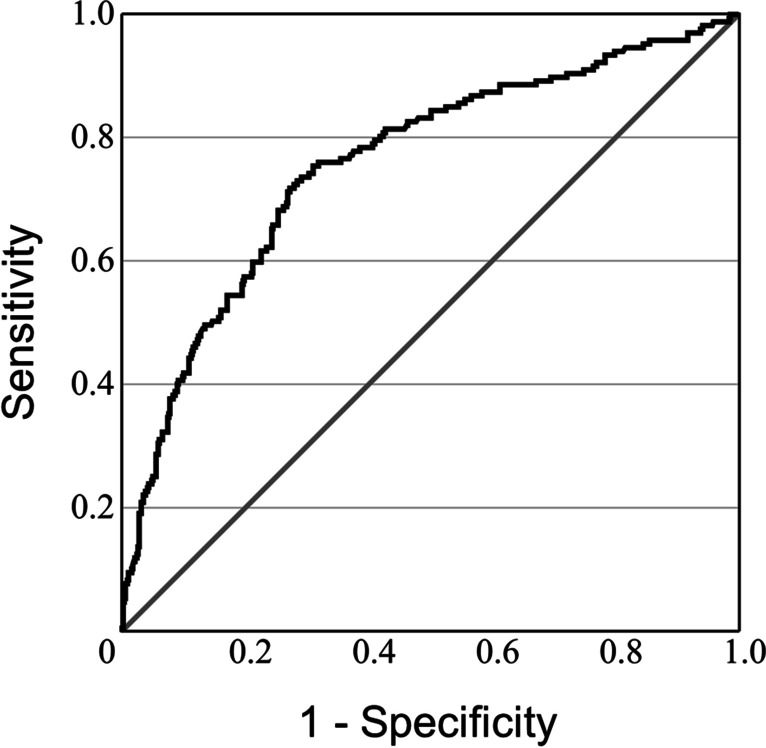
Receiver operating characteristics (ROC) showing the area under the curve (AUC) for “LD-monotherapy” vs. “all other treatments” (see text for details)

ROC analysis revealed an area under the curve of 0.758 (95% CI [0.715, 0.802]) (Fig. 3). Split sample validation showed that the model correctly classified 15/41 patients who received an LD monotherapy, resulting in a sensitivity of 36.6%. The model correctly identified 153/165 PD patients who were prescribed a dopaminergic drug or drug combination other than LD monotherapy, resulting in a specificity of 92.7%. The model’s positive and negative predictive values were 0.586 and 0.838, respectively.

## Discussion

PRISM trial data showed that the prevalence of LD monotherapy differed significantly across European countries. The prescription of LD monotherapy was positively correlated with subject age, and negatively with disease duration. Country of residence was an independent predictor, suggesting that country-specific factors affect prescription practice. The binary logistic model showed excellent specificity but low sensitivity to recognize the treatment condition. Similarly, ROC analyses and split sample validation revealed good specificity, but poor sensitivity to predict treatment class in the PRISM sample.

Younger patients were less likely to be treated with LD monotherapy. A total of 13.1% of PD patients < 60 years of age were treated with LD monotherapy, compared with 25.6% of the study population older than 60 years. This is in line with previous studies reporting preferred use of dopaminergic drugs other than LD in younger individuals with PD [19]. This implies that most prescribers still follow outdated recommendations to avoid LD in younger PD individuals [7]. The long-standing dogma to obviate the use of LD in young early stage PD patients likely is based on previous studies that suggested neurotoxic effects of LD [20], but potential neuroprotective effects of DA and MAOB-I [21, 22]. Both hypotheses have later been refuted [23, 24]. Indeed, recent research suggests that long-term QoL of PD patients is equivalent or even better in individuals treated with LD compared to those receiving MAOB-I or DA [24, 25]. Importantly, disease duration, motor symptom severity, and LD daily dosage rather than the time-point of first LD administration are predictors of LD-induced dyskinesias [26]. These findings have been transferred into recent treatment guidelines, most of which recommend DA and LD as equally suitable to treat symptomatic PD patients no matter of subject age, and even suggest the initial prescription of LD rather than DA to patients with motor symptom related impairment of QoL [8, 9]. However, to thoroughly inform patients about the greater improvement of motor symptoms but increased risk of early motor complications when initially prescribing LD is recommended by all guidelines, and the dose of LD should be kept as low as possible to minimize the risk of dyskinesias. Longer disease duration was associated with a lower likelihood of receiving LD monotherapy. Compared to DA, catechol-O-methyltransferase inhibitors (COMT-I), and MAOB-I, the half-life of immediate release LD is short. The pulsatile stimulation by LD is a main driver of peak-dose dyskinesias [6]. Disease progression inevitably is associated with a decrease of endogenous LD availability, increasing dependence on exogenous LD supplementation and, consequently, motor fluctuations [6]. Thus, it is unsurprising that people with advanced PD received adjunct dopaminergic therapeutics to counteract pulsatile stimulation of dopamine receptors [27]. Interestingly, country of residence was an independent factor predicting LD monotherapy. This is in line with previous studies suggesting widely varying usage of LD monotherapy across countries [10, 19]. For example, the rate of LD monotherapy in the USA has been reported to be around 70% [28, 29], contrasting prescription rates of 14.8% in Italy, 49.6% in Japan, or 29% in the UK [28, 30, 31]. The highest rate of LD monotherapy was observed in the UK at 38%, which was higher than previously reported [28]. Of note, the number of patients younger than 60 years of age was higher in Germany and Spain compared with the other countries. That said, people with PD in the UK did not differ from the other countries in terms of age distribution. Moreover, including country in the multivariate binary logistic model improved model performance (− 2 log likelihood without country = 722.508 vs. including country = 685.747), indicating that country of residence constituted an independent predictor influencing prescription practice. Earlier studies have reported that patients treated by neurologists were more likely to receive multiple medications, whereas the likelihood to be treated with LD monotherapy was higher in PD individuals suffering from multiple comorbidities [29, 32]. People living in rural areas more often receive LD compared to individuals residing in urban areas [33]. Women have been found to be less likely to be treated with multiple medications and tend to initiate LD treatment later than men, likely due to evidence that female gender is a risk factor for the development of dyskinesias [6, 32]. Travel distance to a specialist (but not the number of specialist visits during the past 12 months), out-of-pocket costs, PDQ-39/PD-NMS scores for prescription drugs differed across countries in the PRISM trial data. That said, none of these factors were predictors of prescription patterns. Even though out-of-pocket-costs did not affect the prevalence of LD monotherapy in the study sample, country-specific differences in health care policy, drug pricing, and reimbursement strategies may have influenced drug prescription [34]. Other factors potentially impacting prescription patterns not assessed in the PRISM trial are individual preferences of physicians and patients and economic considerations [35].

ROC analyses and split sample validation provided excellent specificity but poor sensitivity to predict treatment class in the PRISM sample. The poor sensitivity to detect LD monotherapy underscores the existence of additional factors not assessed in the PRISM trial that affect medication patterns, and the usage of LD monotherapy in particular.

There are limitations to our study. PRISM trial data are survey based, and thus are prone to selection bias and inaccuracies. Furthermore, disease severity was not systematically assessed. PD is a progressive neurodegenerative disease. Thus, longer disease duration is expected to be associated with more severe disease symptoms. Nevertheless, individual risk factors and lifestyle do affect disease progression [36], and individual disease courses vary. Thus, disease duration and symptom severity are not necessarily redundant, and the latter may well have influenced prescription patterns in the study sample. Moreover, there were missing data in some categories and the number of patients recruited was not distributed evenly across the six countries, which may have biased analyses. The proportion of PD patients receiving LD in combination with other drugs was much higher than 22%. However, because the variety of drug combinations was large but the number of patients in the respective treatment categories small, we were not able to extend our analyses to other drugs or drug combinations. Lastly, PRISM data do not provide information about the sequence in which drugs were prescribed in patients receiving combination therapies. As a result, the number of patients that initially were started on LD monotherapy may be higher than the number of individuals currently receiving this therapy.

In conclusion, our analyses confirmed prior reports of highly variable prescription patterns to treat PD. Age and disease duration were strong predictors of whether people with PD were treated with LD monotherapy or other dopaminergic drugs or drug combinations. Country of residence was an independent predictor of LD monotherapy. Medical and socio-economic factors such as gender, access to a specialist, presence of comorbidities or dementia, and medication co-pay did not affect prescription patterns, suggesting distinct country-specific socio-demographic factors influencing prescription patterns that were not assessed in the PRISM trial. Ideally, clinical phenotype, severity of motor and non-motor symptoms, patient’s age, risk of adverse effects, personal life circumstances, and needs of PD patients should be considered to determine optimal individual treatment. The small proportion of people with PD below 60 years treated with LD monotherapy indicates that most prescribers still avoid to prescribe LD in young PD individuals.

### Supplementary Information

Below is the link to the electronic supplementary material.

Supplementary file1 (DOCX 13 KB)

## Data Availability

All data used for the preparation of this manuscript were obtained from the previously published Parkinson’s Real World Impact assesSMent (PRISM) study. Data are publicly available after registration at http://www.prism.bial.com.
