# Supplementary material for: Dopaminergic treatment strategies for people with Parkinson’s disease in Europe: a retrospective analysis of PRISM trial data
Source: Neurol Sci. 2023 Jun 13;44(11):3905–12. doi: 10.1007/s10072-023-06888-5 (PMC10570205; doi:10.1007/s10072-023-06888-5)
Supplement: Supplementary file 1 — Supplementary file1 (DOCX 13 KB) [file 10072_2023_6888_MOESM1_ESM.docx]

| **TABLE S1** | |  | |
| --- | --- | --- | --- |
| Category | Missing data N [%] | | |
| Age now  Age at diagnosis  Disease duration  Country of residence  PDQ-39 total score  PD-NMS total score  Presence of comorbidities  Number of comorbidities  Presence of dementia  Specialist appointment in the last 12 months  One-way travel distance between home/specialist  Out of pocket costs for prescription drugs  Emergency room/hospital visit in the last 12 months | | | 6 [0.8 %]  34 [4.3 %]  36 [4.6 %]  0 [0 %]  1 [0.1 %]  205 [26 %]  0 [0 %]  2 [0.3 %]  15 [1.9 %]  75 [9.5 %]  13 [1.7 %]  61 [7.7 %]  161 [20.4 %] |

**Table S1:** Missing data of study sample. PDQ: Parkinson’s disease questionnaire, PD-NMS: Parkinson’s disease non-motor symptoms scale
